# Risk of primary lung cancer after adjuvant radiotherapy in breast cancer—a large population-based study

**DOI:** 10.1038/s41523-021-00280-2

**Published:** 2021-06-01

**Authors:** Anna-Karin Wennstig, Charlotta Wadsten, Hans Garmo, Mikael Johansson, Irma Fredriksson, Carl Blomqvist, Lars Holmberg, Greger Nilsson, Malin Sund

**Affiliations:** 1 Department of Surgical and Perioperative Sciences, Umeå University, Umeå, Sweden; 2 Department of Oncology, Sundsvall Hospital, Sundsvall, Sweden; 3 Department of Surgery, Sundsvall Hospital, Sundsvall, Sweden; 4 Regional Cancer Center, Uppsala University/ Uppsala University Hospital, Uppsala, Sweden; 5 Department of Radiation Sciences, Umeå University, Umeå, Sweden; 6 Department of Breast-and Endocrine Surgery, Karolinska University Hospital, Stockholm, Sweden; 7 Department of Molecular Medicine and Surgery, Karolinska Institutet, Stockholm, Sweden; 8 Department of Oncology, Örebro University, University Hospital, Örebro, Sweden; 9 Translational Oncology & Urology Research (TOUR), School of Cancer and Pharmaceutical Sciences, King’s College London, London, UK; 10 Department of Surgical Sciences, Uppsala University, Uppsala, Sweden; 11 Department of Immunology, Genetics and Pathology, Section of Experimental and Clinical Oncology, Uppsala University, University Hospital, Uppsala, Sweden; 12 Department of Oncology, Gävle Hospital, Gävle, Sweden; 13 Department of Oncology, Visby Hospital, Visby, Sweden

**Keywords:** Breast cancer, Radiotherapy

## Abstract

Adjuvant radiotherapy (RT) for breast cancer (BC) has been associated with an increased risk of later radiation-induced lung cancer (LC). We examined the risk of primary LC in a population-based cohort of 52300 women treated for BC during 1992 to 2012, and 253796 age-matched women without BC. Cumulative incidence of LC was calculated by the Kaplan–Meier method, and the risk of LC after BC treatment was estimated by Cox proportional hazards regression analyses. Women with BC receiving RT had a higher cumulative incidence of LC compared to women with BC not receiving RT and women without BC. This became apparent 5 years after RT and increased with longer follow-up. Women with BC receiving RT had a Hazard ratio of 1.59 (95% confidence interval 1.37–1.84) for LC compared to women without BC. RT techniques that lower the incidental lung doses, e.g breathing adaption techniques, may lower this risk.

## Introduction

Long-term side effects of breast cancer (BC) treatment and quality of life for BC survivors have become an issue of great importance with improved prognosis and increasing BC incidence rates leading to a high prevalence of BC survivors^1^. In adjuvant postoperative BC radiotherapy (RT), the main long-term hazards are ischemic heart disease and lung cancer (LC)^2,3^.

Several studies show an increased risk of LC after adjuvant BC RT^4–12^. Most published studies include women treated with older radiation regimens with techniques and dosages no longer in clinical use, and it is uncertain whether these results may be applied on more contemporary RT regimens, however, a few studies report an increased risk of primary LC after BC diagnosis also in women receiving RT in the 1990s and 2000s^13–15^.

In addition to RT, many women receive adjuvant endocrine therapy and chemotherapy, and some of these agents also are related to a higher risk of second malignancies^16–18^. Tamoxifen, on the other hand, has been suggested to reduce the risk of LC after BC diagnosis^19^. Another study, however, has reported opposite results^20^.

Tobacco smoking is a major risk factor for LC and some studies show a strong interaction between RT and smoking on the risk of later LC^3,21–25^.

The aim of the present study was to examine the risk of LC in women who received adjuvant RT for BC during 1992 to 2012 and in age-matched women without a history of BC. The possible modulation of the LC risk by adding endocrine therapy and chemotherapy was also studied.

## Results

### Study population

The study population consisted of 52,300 women with BC and 253,796 women without a history of BC (Supplementary Table 1). The mean follow-up time was 7.9 years in women with BC and 8.8 years in women without BC. Women with BC had a higher educational level compared to women without BC diagnosis; 28.4% of the women with BC had an education longer than 12 years compared to 25.2% of the women without BC. No major differences were seen between the women with BC and women without BC concerning CCI, and the vast majority of women in the study population had no reported comorbidity.

Patient characteristics for women with BC are shown in Supplementary Table 2. The group was stratified by receiving RT or not. Women not receiving RT were generally older and had more comorbidities according to CCI. They were more often selected for mastectomy and less frequently selected for systemic therapy compared to women receiving RT. Characteristics of women diagnosed with LC within the first year from BC diagnosis are shown in Supplementary Table 3. The ORs increased with age and with more comorbidities. A higher OR for LC diagnosis within the first year of BC diagnosis was also seen in women diagnosed in the latter part of the inclusion period.

### Cumulative incidence of LC

**Fig. 1:**
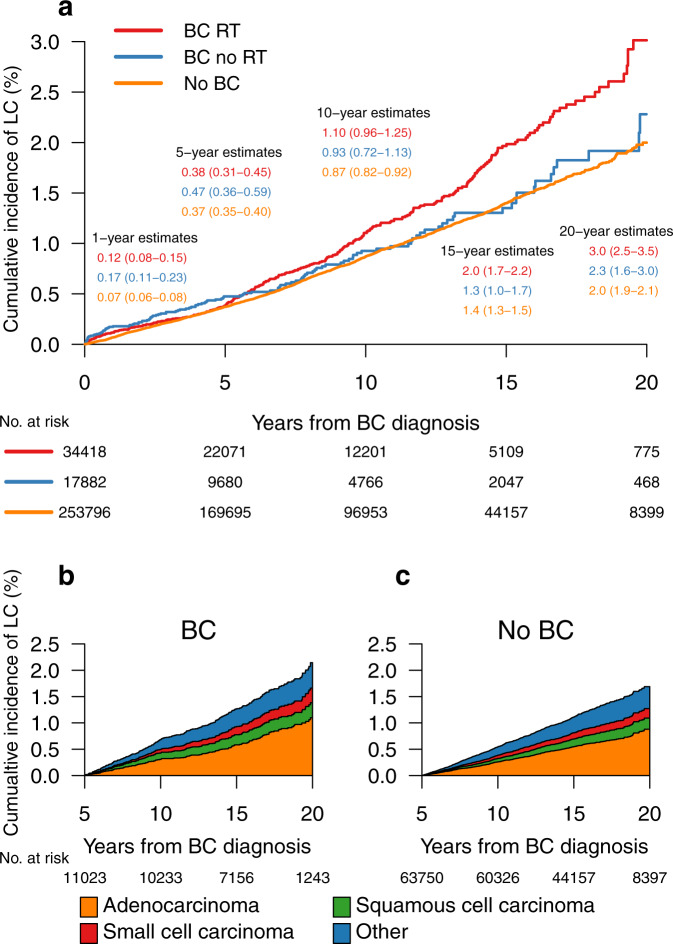
Cumulative incidence of lung cancer. Cumulative incidence of lung cancer (LC) for women without breast cancer (BC) diagnosis, women with BC not receiving radiotherapy (RT), and women with BC receiving RT (**a**). The distribution of the different LC histological subtypes for women with follow-up of longer than five years is displayed in (**b**) for women with BC, and in (**c**) for women without BC diagnosis. Number (No.).

The cumulative incidence of LC is visualized in Fig. 1a. Women with follow-up of longer than five years were also stratified by histological type of LC, and the distribution of different histological subtypes was similar for women with BC and women without BC (Fig. 1b and c). An increased cumulative incidence of LC in women with BC receiving RT was evident at ten-year follow-up. The LC incidence continued to increase throughout the follow-up time, showing cumulative rates of 3.0%, 2.3%, and 2.0%, for women with BC receiving RT, women with BC not receiving RT, and women without BC diagnosis, respectively, at 20-year follow-up. The LC incidence was separately analyzed for women <50 years of age and those ≥50 years of age. The results were similar in both groups although the number of cases was low in the younger age group (data not shown).

### Adjusted risk of LC in women with follow-up times of more than 5 years

The risk of LC in women with BC who had an event-free survival of longer than five years, compared to women without BC diagnosis, is shown in Table 1. The risk of LC was higher in women with BC compared to women without a BC diagnosis, with an adjusted hazard ratio (HR) of 1.35 (95% confidence interval (CI) 1.18–1.54). In women with BC who did not receive RT no significant difference in risk of LC was seen compared to women without BC (HR 0.81, 95% CI 0.62–1.08). Women with BC who received RT had a higher risk of LC compared to women without BC, with a HR of 1.59 (95% CI 1.37–1.84). Subgroup analyses according to pathological lymph node stage showed HRs of 1.41 (95% CI 1.20–1.65), 1.29 (95% CI 0.97–1.70), and 1.01 (95% CI 0.58–1.75), for N0, N1-3, and N4+, respectively, compared to women without BC. When women receiving RT were stratified by pathological lymph node stage, the HRs for LC were 1.62 (95% CI 1.35–1.94), 1.48 (95% CI 1.10–1.99), and 1.15 (95% CI 0.66–1.98) for N0, N1-3, and N4+, respectively, compared to women without BC. When women were stratified for other adjuvant therapies, a HR of 1.33 (95% CI 1.09–1.62) was seen in women not selected for endocrine therapy, and of 1.35 (95% CI 1.15–1.60) in women selected for endocrine therapy, compared to women without BC. Analyses concerning chemotherapy showed a HR of 1.34 (95% CI 1.16–1.56) and of 1.35 (95% CI 1.02–1.78), in women not selected for chemotherapy and women selected for chemotherapy, respectively.

**Table 1:** Risk of lung cancer in women with breast cancer compared to women without breast cancer diagnosis.

	No. of events	Incidence per 100,000 pyrs	Crude		Adjusted^a^	
			HR	95% CI	HR	95% CI
*BC*						
No BC	976	103.8	1.00	Ref.	1.00	Ref.
BC	270	138.6	1.34	(1.17–1.54)	1.35	(1.18–1.54)
*RT*						
No BC	976	103.8	1.00	Ref.	1.00	Ref.
BC, No RT	52	94.0	0.91	(0.69–1.20)	0.81	(0.62–1.08)
BC, RT	218	156.2	1.52	(1.31–1.76)	1.59	(1.37–1.84)
*N-stage*						
No BC	976	103.8	1.00	Ref.	1.00	Ref.
BC N0	181	145.6	1.41	(1.20–1.65)	1.41	(1.20–1.65)
BC N1-3	52	126.4	1.23	(0.93–1.63)	1.29	(0.97–1.70)
BC N4+	13	95.5	0.94	(0.54–1.62)	1.01	(0.58–1.75)
BC NX	24	151.8	1.49	(0.99–2.24)	1.28	(0.85–1.92)
*RT/N-stage*						
No BC	976	103.8	1.00	Ref.	1.00	Ref.
BC, No RT	52	94.0	0.91	(0.69–1.20)	0.82	(0.62–1.08)
BC, RT N0	136	160.2	1.55	(1.29–1.85)	1.62	(1.35–1.94)
BC, RT N1-3	46	141.9	1.38	(1.03–1.86)	1.48	(1.10–1.99)
BC, RT N4+	13	106.6	1.04	(0.60–1.81)	1.15	(0.66–1.98)
BC, RT NX	23	229.0	2.24	(1.48–3.39)	2.15	(1.42–3.25)
*ER-status*						
No BC	976	103.8	1.00	Ref.	1.00	Ref.
BC, ER+	168	135.0	1.32	(1.12–1.56)	1.28	(1.09–1.51)
BC, ER−	45	149.5	1.44	(1.07–1.94)	1.54	(1.14–2.08)
BC, ER missing	57	141.3	1.34	(1.02–1.75)	1.41	(1.08–1.85)
*PR-status*						
No BC	976	103.8	1.00	Ref.	1.00	Ref.
BC, PR+	125	115.1	1.12	(0.93–1.35)	1.11	(0.92–1.34)
BC, PR-	85	191.2	1.87	(1.49–2.33)	1.83	(1.47–2.29)
BC, PR missing	60	143.6	1.36	(1.05–1.77)	1.43	(1.10–1.86)
*Endocrine therapy*						
No BC	976	103.8	1.00	Ref.	1.00	Ref.
BC, No Endo	109	132.4	1.26	(1.04–1.54)	1.33	(1.09–1.62)
BC, Endo	161	143.1	1.40	(1.19–1.66)	1.35	(1.15–1.60)
*Chemotherapy*						
No BC	976	103.8	1.00	Ref.	1.00	Ref.
BC, No Chemo	217	146.6	1.41	(1.22–1.64)	1.34	(1.16–1.56)
BC, Chemo	53	113.1	1.11	(0.84–1.47)	1.35	(1.02–1.78)

Hazard ratios (HR) for lung cancer (LC) conditioned on 5-year event-free survival. Restricted to women undergoing surgery for breast cancer (BC) and their comparison women without BC diagnosis. Number (No.), incidence (Inc.), person years (pyrs), reference (Ref.) radiotherapy (RT), pathological lymph node stage (N-status), estrogen receptor status (ER-status), progesterone receptor status (PR-status), endocrine therapy (endo), chemotherapy (chemo), and Charlson comorbidity index (CCI).

^a^Adjusted for age, year of BC, educational level, chronic pulmonary disease (CPD), and CCI except CPD.

The analyses were performed stratified by follow-up time, for women with event-free survival of 7.5, 10, 12.5, and 15, years respectively (data not shown). A similar pattern was seen in these subgroups as for the whole group of women with an event-free survival of longer than five years, though there were fewer events in the subgroups, and overall the HRs did not reach statistically significance.

### Adjusted risk of LC for women with BC by laterality

The risk of ipsilateral and contralateral LC, in relation to the laterality of the breast tumor, for women with BC who had an event-free survival of longer than 5 years, compared to women without BC diagnosis, is shown in Table 2. The adjusted risk of ipsilateral LC was 1.82 (95% CI 1.47–2.25) in women with BC receiving RT compared to women without BC diagnosis, and 1.55 (95% CI 1.25–1.93) for contralateral LC in women receiving RT compared to women without BC diagnosis. When women receiving RT were stratified by pathological lymph node stage the HRs for an ipsilateral LC were 1.92 (95% CI 1.49–2.47), 1.72 (95% CI 1.13–2.61), and 1.64 (95% CI 0.81–3.30), for N0, N1-3, and N4+, respectively, compared to women without BC diagnosis. The HRs for a contralateral LC was 1.50 (95% CI 1.14–1.96), 1.51 (95% CI 0.98–2.31), and 0.93 (95% CI 0.39–2.26), for N0, N1-3, and N4+, respectively, compared to women without BC diagnosis. For women diagnosed with BC between 1992 and 2001, the adjusted risk of ipsilateral LC was 1.92 (95% CI 1.51–2.43) in women with BC receiving RT compared to women without BC diagnosis, and 1.49 (95% CI 1.15–1.93) for contralateral LC in women receiving RT compared to women without BC diagnosis (Supplementary Table 4).

**Table 2:** Risk of ipsi- and contralateral lung cancer in women with breast cancer compared to women without breast cancer diagnosis.

	No. of events	Incidence per 100,000 pyrs	Crude		Adjusted^a^	
			HR	95% CI	HR	95% CI
*Ipsilateral LC*						
BC						
No BC	420	46.2	1.00	Ref.	1.00	Ref.
BC	134	71.1	1.55	(1.28–1.88)	1.55	(1.27–1.88)
*RT*						
No BC	420	46.2	1.00	Ref.	1.00	Ref.
BC, No RT	26	49.2	1.07	(0.72–1.59)	0.96	(0.64–1.42)
BC, RT	108	79.7	1.74	(1.41–2.15)	1.82	(1.47–2.25)
*RT/N-stage*						
No BC	420	46.2	1.00	Ref.	1.00	Ref.
BC, No RT	26	49.2	1.07	(0.72–1.59)	0.96	(0.64–1.42)
BC, RT N0	70	84.9	1.85	(1.43–2.38)	1.92	(1.49–2.47)
BC, RT N1-3	23	73.2	1.60	(1.05–2.44)	1.72	(1.13–2.61)
BC, RT N4+	8	67.3	1.49	(0.74–2.99)	1.64	(0.81–3.30)
BC, RT NX	7	71.3	1.57	(0.74–3.31)	1.50	(0.71–3.16)
*Contralateral LC*						
BC						
No BC	456	50.2	1.00	Ref.	1.00	Ref.
BC	122	64.7	1.30	(1.06–1.58)	1.31	(1.07–1.59)
*RT*						
No BC	456	50.2	1.00	Ref.	1.00	Ref.
BC, No RT	22	41.6	0.83	(0.54–1.27)	0.75	(0.49–1.16)
BC, RT	100	73.8	1.48	(1.19–1.84)	1.55	(1.25–1.93)
*RT/N-stage*						
No BC	456	50.2	1.00	Ref.	1.00	Ref.
BC, No RT	22	41.6	0.83	(0.54–1.27)	0.76	(0.49–1.16)
BC, RT N0	59	71.6	1.43	(1.09–1.88)	1.50	(1.14–1.96)
BC, RT N1-3	22	70.0	1.41	(0.92–2.16)	1.51	(0.98–2.31)
BC, RT N4+	5	42.1	0.85	(0.35–2.06)	0.93	(0.39–2.26)
BC, RT NX	14	142.6	2.90	(1.70–4.93)	2.77	(1.63–4.73)

Hazard ratios (HR) for ipsi- and contralateral lung cancer (LC) conditioned on 5-year event-free survival. Restricted to women undergoing surgery for breast cancer (BC) and their comparison women without BC diagnosis, Number (No.), incidence (Inc.), person years (pyrs), reference (Ref.), and radiotherapy (RT).

^a^Adjusted for age, year of BC, educational level, chronic pulmonary disease (CPD), and CCI except CPD.

### Lung cancer-specific survival

**Fig. 2:**
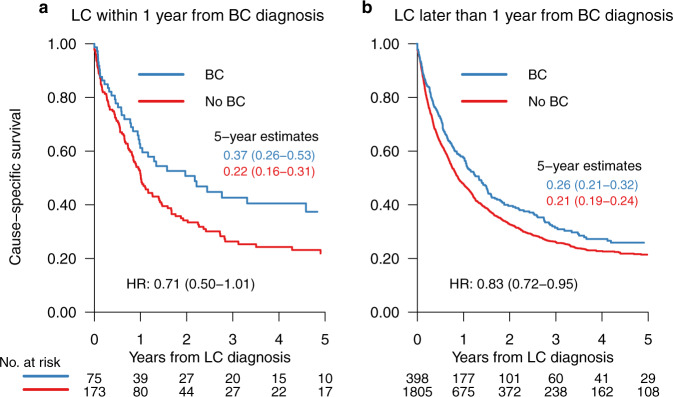
Lung cancer-specific survival. Lung cancer-specific survival within (**a**) and after (**b**) the first year from study onset, for women with breast cancer (BC) and women without BC diagnosis. Lung cancer (LC) and number (No).

The LC-specific survival for women with BC and women without BC, stratified by LC diagnosis within or after the first year from BC diagnosis, is shown in Fig. 2. The HRs for LC-specific survival in women with BC compared to women without BC diagnosis was 0.71 (95% CI 0.50–1.01), and 0.83 (95% CI 0.72–0.95) for the two strata of time since BC diagnosis. The five-year LC-specific survival was 37% for women with BC diagnosed with LC within the first year from BC diagnosis compared to 22% for women without BC (Fig. 2a). The corresponding numbers were 26% for women with BC compared to 21% for women without BC, for women diagnosed with LC after the first year from BC diagnosis (Fig. 2b).

## Discussion

We found an increased risk of primary LC after BC diagnosis in women receiving adjuvant RT for BC from 1992 to 2012 compared to age-matched women without BC, with a HR of 1.59 (95% CI 1.37–1.84). The cumulative incidence of LC was 3.0%, 2.3%, and 2.0%, for women with BC receiving RT, women with BC not receiving RT, and women without BC diagnosis, respectively, at 20-year follow-up. The curves started to deviate at five years from BC diagnosis and the observed difference in incidence continued to increase at longer follow-up.

A strength of this study is the population-based setting and the large size of the cohort. Through linkage with other registers we were able to adjust for comorbidity and educational level. A limitation with the study is the relative short follow-up time. Women with BC had a median follow-up time of 8.2 years, which is short regarding the risk of radiation-induced LC for women receiving RT during the later part of the inclusion period. A further limitation is the lack of information on smoking status, which would have been of interest as previous studies suggest that radiation-induced LC in women with BC mainly occurs in smokers^3,21^. We also lacked information on other factors that may be associated with LC, such as alcohol use, body mass index, and genetic predisposition. Information concerning individual radiation doses and targets was not available in the study. The inclusion time of the study corresponded with the implementation of computed tomography-based RT (3DCRT) planning in Sweden, and most of the women in the study have likely received 3DCRT given with tangential fields to the breast or chest wall, still considered as standard RT in many Swedish RT departments. Most women with lymph node metastases also received RT to the axilla, the supraclavicular fossa, and in some cases the internal mammary chain (IMC).

Several previous studies show an increased risk of radiation-induced LC^5,7,13^, Most of these studies included patients treated in the 1970s and 1980s with outdated RT techniques and dosages. In the present study we could show that the increased risk of LC after BC irradiation persisted although RT was delivered with more contemporary RT techniques. In agreement with a recent meta-analysis by Grantzau et al.^5^, an increasing cumulative incidence of LC in women receiving RT was evident after five years from BC diagnosis, and increased over time. The study by Grantzau et al.^5^ showed a standardized incidence ratio for LC of 1.91 (95%CI, 1.11–3.29) at ≥15|years after BC diagnosis. A higher incidence of LC was also seen in women with BC the first year from BC diagnosis, suggesting that women with BC were more frequently examined in connection to BC diagnosis, a pattern also described in studies concerning the implementation of cancer screening programs^26^. Women with BC in the present study had better LC-specific survival compared to women without BC diagnosis, especially if diagnosed within the first year from BC diagnosis. LC diagnosis within the first year from BC diagnosis was more common at an older age, and in women with more comorbidities. This is likely due to these women being subjected to more frequent radiological investigations, when presenting with symptoms. LC diagnosis within one year from BC diagnosis was more frequent during the latter part of the inclusion time, suggesting a change in management of women with previous BC diagnosis, e.g., more frequent and accurate investigation techniques used in symptomatic patients. A similar finding is also described in a study by Wang et al.^27^, and thus LC diagnosis at earlier stages could explain the better outcome.

At BC RT, it is mainly the ipsilateral lung that receives radiation dose. In a systematic review of lung doses reported in RT trials from 2010 to 2015 by Aznar et al.^28^, the mean lung dose (MLD) was 8.4 Gray (Gy) to the ipsilateral lung and 0.8 Gy to the contralateral lung in tangential irradiation of the breast or chest wall. The lung doses increased when regional lymph nodes were included in the target, especially the IMC, and in intensity-modulated RT (IMRT)^28^. In a large population-based study, the mortality ratio, ipsilateral vs. contralateral LC, was increased in women irradiated for BC. This was mainly seen in women treated in the 1970s and 1980s, and in women treated in the 1990s and 2000s no significant increase in mortality ratio of ipsilateral LC vs. contralateral LC was seen^7^. In a study conducted in the same population, the relative risk of LC was estimated in women irradiated for BC, based on comparison to the expected numbers of LC in the general population. The relative risk was higher for ipsilateral LC than for contralateral LC in women irradiated for BC. A significantly increased risk of contralateral LC was, however, also seen in women irradiated for BC compared to the general population^4^. In the present study the HRs of ipsilateral and contralateral LC in women receiving RT compared to women without BC diagnosis, was ~1.82 and 1.55, respectively. When the analysis was restricted to women treated between 1992 and 2001, the HRs were 1.92 and 1.49, respectively. A tendency to offer women with previous BC more extensive examinations may contribute to the increase in the risk of LC, and may be one explanation for the increase in the risk of contralateral LC seen in women receiving RT. The LC incidence is higher in the right lung than the left lung based on asymmetries in organ size, and likewise the incidence of left-sided BC is higher than that of right-sided BC^29^. This disparity may also affect analyses based on the laterality of the lung or breast.

Smoking is strongly related to the risk of LC^30^. Taylor et al.^3^ estimated that a 50-year old smoker receiving a MLD of 5 Gy for BC RT, would have an absolute increase in the risk of radiation-induced LC of 4% at the age of 80, and a non-smoker would have an absolute increase in risk of 0.3%. Unfortunately, smoking status was not available in the present study. The smoking habits have changed over the last decades, and the reported smoking incidence in Swedish women have decreased from 27% in the late 1980s to 7% during 2018^31^. Since smoking habits are related to socioeconomic factors, like educational level^39^, and women with BC in this study had a higher level of education compared to women without BC, there may be fewer smokers among women with BC than the women without BC.

Breathing adaption techniques are currently used mainly in left-sided irradiation in order to lower cardiac doses but may be used in a wider extent to decrease lung doses as well^28^. Implementation of other RT techniques that are shown to reduce lung doses, like prone patient positioning or proton therapy, may lower the risk of LC after BC diagnosis, and for patients with low risk of BC recurrence, partial breast irradiation or omitting RT may be an option.

In conclusion, we found an increase in LC after BC diagnosis in women receiving RT for BC with contemporary RT techniques during 1992 to 2012. Implementation of RT techniques and regimens that lower the lung doses must be emphasized to reduce the risk of secondary malignancies. Relevant information to patients regarding risks of radiation-induced LC related to smoking and smoking cessation support is of utmost importance.

## Methods

### Study population

Information regarding histopathological data and BC treatments on Swedish women with BC is recorded in regional BC registries since the seventies, and since 2008 all BC patients are registered in the National Quality Register for Breast Cancer (INCA). The registers from three of Sweden’s six health care regions (Stockholm, Uppsala-Örebro, and the Northern region) were merged to form the Breast Cancer DataBase Sweden (BCBaSe) cohort. All new cases of invasive BC in women from 1992 to 2012 are included, and five age-matched women without a BC diagnosis, are added for each BC case to form a comparison cohort. For the present study all women with early BC were selected from the BCBaSe cohort, and women with metastatic BC at diagnosis, a contralateral BC during follow-up, or a history of another malignancy were excluded. For the Northern region, only women treated between 2008 and 2012 were included, due to inconsistencies concerning registration in the regional BC register prior to 2008.

By using the unique personal identity numbers issued to all Swedish citizens, the study cohort was linked to the National Patient Register (NPR), Swedish Cancer Register, Cause of Death register, and the Longitudinal Integration Database for Health Insurance and Labor Market Studies. NPR is a validated register that contains records of all hospital discharges since 1987, and hospital-based outpatient care since 2001. The NPR contains information on main diagnosis and up to eight secondary diagnoses^32^. Comorbidity was classified using the NPR according to The Charlson Comorbidity Index (CCI) in three comorbidity levels: CCI 0 (no comorbidity), CCI 1 (mild comorbidity), and CCI 2 (severe comorbidity)^33^.

LC was identified in the Swedish Cancer Register. The Swedish Cancer Register was founded in 1958 and comprises information on type of cancer diagnosis according to International Classification of Diseases (ICD) codes. From 2005 and onwards, SNOMED tumor morphological codes are included in the Swedish Cancer Register^34,35^.

Cause of death and date of death were retrieved from the Cause of Death register^36^, and in case of emigration, the date of emigration was identified in the Total Population Register^37^. The Longitudinal Integration Database for Health Insurance and Labor Market Studies contains data from the labor market, the educational, and social sectors^38^. This registry contains data on socioeconomic variables, such as highest level of education, place of employment, income, and marital status.

### Statistical methods

LC was defined according to the Classification of Disease (ICD) 9th edition codes 162.0-162.9 or ICD-10 codes C34.0-34.9 and categorized into four groups based on LC histology: adenocarcinoma, squamous cell cancer, small cell LC, and others (Supplementary Table 5).

The women were followed from the date of inclusion to date of LC, death, emigration, or end of follow-up (31st of December 2013) whichever came first, censoring for death without previous LC, emigration, and end of follow-up. Odds ratios (OR) of LC within one year from BC diagnosis were modeled through logistic regression.

Cumulative incidence of LC was calculated by the Kaplan-Meier method for women with BC and for the women without BC diagnosis. To analyze the subtypes of LC, a left truncation on December the 31st 2004 was performed, due to the start of SNOMED registration. Stacked cumulative incidences of subtypes of LC were used, where the different subtypes of LC were considered as competing events.

To calculate the risk of LC, Cox proportional hazard regression analyses were performed.

Analyses were performed separately conditioned on 5, 7.5, 10, 12.5, and 15-year event-free survival of the BC index case and were restricted to women with BC who underwent BC surgery and their comparison women without BC diagnosis. The analyses were stratified for RT, pathological lymph node stage, endocrine therapy, chemotherapy, and of BC tumor characteristics including estrogen (ER) and progesterone receptor (PR) status.

The analyses were adjusted for age, educational level (low: <10 years mandatory school, intermediate: 10–12 years high school, and high: university or college), year of BC diagnosis, chronic pulmonary disease (CPD), and CCI except CPD.

To calculate the risk of ipsilateral and contralateral LC, Cox proportional hazard regression analyses were performed. BC laterality for the comparison women without BC was set to be the same as the matched women with BC. Women with bilateral BC and their comparison women were excluded. The laterality analyses were censored for LC with unknown laterality. In analyses of ipsilateral LC, contralateral LC was censored and vice versa for the analyses of contralateral LC. A separate analysis was performed for women diagnosed with BC between 1992 and 2001.

To assess LC-specific survival, Kaplan-Meier analyses were performed separately for women diagnosed with LC within or after the first year from BC diagnosis.

Women diagnosed with LC were followed from date of LC diagnosis to date of death, or end of follow-up. In this analysis death from other causes was censored and death with LC as the main cause of death was considered an event.

All analyses were performed using the statistical software R^39^.

### Ethics approvals

All procedures performed in this study involving human participants were in accordance with the ethical standards of the regional committee of ethics in Stockholm, reference number 2013/1272-31/4, and with the 164 Helsinki declaration and its later amendments or comparable ethical standards. The cohort is based on data from regional and national registries and the need for informed consent was waived.

### Reporting summary

Further information on research design is available in the Nature Research Reporting Summary linked to this article.

## Supplementary information

Supplementary Information

Reporting Summary

## Data Availability

The data generated and analyzed during this study are described in the following data record: 10.6084/m9.figshare.14541423^40^. The Breast Cancer DataBase Sweden (BCBaSe) cohort was used in this study. It is a population-based database that comprises all new cases of invasive breast cancer in women from 1992 to 2012 in three Swedish health care regions, and a comparison cohort of five age-matched women without a breast cancer diagnosis for each case of breast cancer. The cohort was linked to a number of national population-based registries. Since BCBaSe contains sensitive health information, it cannot be published in open repositories. Those interested in data from BCBaSe should contact the chairman of the steering committee of the cohort, Malin Sund (malin.sund@umu.se). The estimates of cumulative incidence of lung cancer and risk of lung cancer after breast cancer treatment data are contained in the file ‘LCa_20191204.csv’ which is housed on institutional storage. These data are not publicly available for the following reason: data contain sensitive health information. However, the data can be made available upon request to the corresponding author.

## Code availability

The code is available from the corresponding author upon reasonable request. Data management was performed in SAS version 14.2 Copyright (c) 2002-2012 by SAS Institute Inc., Cary, NC, USA. Statistical analysis was performed with R, version 3.6.1 24. Team R.C.: R: a language and environment for statistical computing. 2013. R Foundation for Statistical Computing, Vienna.
