# Supplementary material for: Risk of primary lung cancer after adjuvant radiotherapy in breast cancer—a large population-based study
Source: NPJ Breast Cancer. 2021 Jun 1;7:71. doi: 10.1038/s41523-021-00280-2 (PMC8169889; doi:10.1038/s41523-021-00280-2)
Supplement: Supplementary file 1 — Supplementary Information [file 41523_2021_280_MOESM1_ESM.pdf]

Supplementary information

Supplementary Table 1: Characteristics of the study population

	BC (n=52,300)		No BC (n=253,796)		Total (n=306,096)	
**FU-time, mean (sd), yrs**	7.9	(5.5)	8.8	(5.6)	8.6	(5.6)
**Health care region, n (%)**						
Stockholm	24,740	(47.3)	120,164	(47.3)	144,904	(47.3)
Uppsala/Örebro	24,305	(46.5)	117,922	(46.5)	142,227	(46.5)
Northern	3,255	(6.2)	15,710	(6.2)	18,965	(6.2)
**Year of BC diagnosis, n (%)**						
1992-1997	11,801	(22.6)	57,529	(22.7)	69,330	(22.6)
1998-2002	11,660	(22.3)	56,739	(22.4)	68,399	(22.3)
2003-2007	12,684	(24.3)	61,527	(24.2)	74,211	(24.2)
2008-2012	16,155	(30.9)	78,001	(30.7)	94,156	(30.8)
**Age at study start, n (%)**						
<45 yrs	5,081	(9.7)	25,237	(9.9)	30,318	(9.9)
45-54 yrs	11,147	(21.3)	55,137	(21.7)	66,284	(21.7)
55-64 yrs	13,742	(26.3)	66,906	(26.4)	80,648	(26.3)
65-74 yrs	11,625	(22.2)	55,800	(22.0)	67,425	(22.0)
75-84 yrs	7,405	(14.2)	35,146	(13.8)	42,551	(13.9)
85+ yrs	3,287	(6.3)	15,570	(6.1)	18,857	(6.2)
**Marital Status, n (%)**						
Married	26,274	(50.2)	127,223	(50.1)	153,497	(50.1)
Not married	26,026	(49.8)	126,573	(49.9)	152,599	(49.9)
**Level of education, n (%)**						
low	15,974	(30.5)	83,128	(32.8)	99,102	(32.4)
middle	19,571	(37.4)	96,096	(37.9)	115,667	(37.8)
high	14,839	(28.4)	63,841	(25.2)	78,680	(25.7)
missing	1,916	(3.7)	10,731	(4.2)	12,647	(4.1)
**CCI, n (%)**						
0	45,631	(87.2)	221,969	(87.5)	267,600	(87.4)
1	4,368	(8.4)	21,569	(8.5)	25,937	(8.5)
2	1,380	(2.6)	6,489	(2.6)	7,869	(2.6)
3+	921	(1.8)	3,769	(1.5)	4,690	(1.5)

Baseline characteristics of women with breast cancer (BC) and women without BC diagnosis. Follow-up (FU), standard deviation (sd), number (n), years (yrs), and Charlson Comborbidity Index (CCI).

Supplementary Table 2: Characteristics of women with breast cancer

	RT (n=34,418)		No RT (n=7,882)		Total (n=52,300)	
**Age, n (%)**						
≤50 yrs	9,014	(26.2)	2,526	(14.1)	11,540	(22.1)
51-60 yrs	10,168	(29.5)	2,542	(14.2)	12,710	(24.3)
61-70 yrs	10,028	(29.1)	3,262	(18.2)	13,290	(25.4)
71-80 yrs	4,485	(13.0)	4,171	(23.3)	8,656	(16.6)
81+ yrs	723	(2.1)	5,381	(30.1)	6,104	(11.7)
**T-stage, n (%)**						
T0	5,383	(15.6)	1,673	(9.4)	7,056	(13.5)
T1	17,693	(51.4)	6,984	(39.1)	24,677	(47.2)
T2	9,128	(26.5)	7,058	(39.5)	16,186	(30.9)
T3	1,525	(4.4)	1,072	(6.0)	2,597	(5.0)
T4	403	(1.2)	661	(3.7)	1,064	(2.0)
TX	286	(0.8)	434	(2.4)	720	(1.4)
**N-stage, n (%)**						
N0	19,764	(57.4)	9,576	(53.6)	29,340	(56.1)
N1-3	8,593	(25.0)	2,653	(14.8)	11,246	(21.5)
N4+	4,433	(12.9)	781	(4.4)	5,214	(10.0)
Not recorded	1,628	(4.7)	4,872	(27.2)	6,500	(12.4)
**ER-status, n (%)**						
ER+	25,292	(73.5)	11,290	(63.1)	36,582	(69.9)
ER-	5,695	(16.5)	2,305	(12.9)	8,000	(15.3)
Missing	3,431	(10.0)	4,287	(24.0)	7,718	(14.8)
**PR-status, n (%)**						
PR+	21,275	(61.8)	9,319	(52.1)	30,594	(58.5)
PR-	9,503	(27.6)	4,117	(23.0)	13,620	(26.0)
Missing	3,640	(10.6)	4,446	(24.9)	8,086	(15.5)
**Surgery, n (%)**						
No surgery	27	(0.1)	2,137	(12.0)	2,164	(4.1)
BCS	25,029	(72.7)	3,258	(18.2)	28,287	(54.1)
Mastectomy	9,245	(26.9)	11,912	(66.6)	21,157	(40.5)
Missing	117	(0.3)	575	(3.2)	692	(1.3)
**BC laterality, n (%)**						
Left	17,470	(50.8)	9,332	(52.2)	26,802	(51.2)
Right	16,670	(48.4)	8,372	(46.8)	25,042	(47.9)
UNS	278	(0.8)	178	(1.0)	456	(0.9)

**Endocrine therapy, n (%)**						
Yes	23,340	(67.8)	10,203	(57.1)	33,543	(64.1)
No	11,078	(32.2)	7,679	(42.9)	18,757	(35.9)
**Chemotherapy, n (%)**						
Yes	12,208	(35.5)	2,699	(15.1)	14,907	(28.5)
No	22,210	(64.5)	15,183	(84.9)	37,393	(71.5)
**Trastuzumab, n (%)** #						
Yes	1,503	(9.0)	375	(5.1)	1,878	(7.8)
No	15,240	(91.0)	6,942	(94.9)	22,182	(92.2)
**CCI, n (%)**						
0	31,800	(92.4)	13,831	(77.3)	45,631	(87.2)
1	1,970	(5.7)	2,398	(13.4)	4,368	(8.4)
2	427	(1.2)	953	(5.3)	1,380	(2.6)
3+	221	(0.6)	700	(3.9)	921	(1.8)

Baseline characteristics for women with breast cancer (BC) stratified by radiotherapy (RT). Number (n), years (yrs), tumor status (T-stage), pathological lymph node stage (N-status), estrogen receptor status (ER-status), progesterone receptor status (PR-status), and Charlson comorbidity index (CCI). #Only for women diagnosed 2005-2012.

Supplementary Table 3: Odds ratio of women with lung cancer diagnosed within one year from breast cancer diagnosis

	No. of LC within 1 year from BC	Odds	OR	95% CI
**Age**				
≤50 yrs	12	0.00017	1.00	Ref.
51-60 yrs	45	0.00060	3.43	(1.88-6.79)
61-70 yrs	89	0.00115	6.60	(3.76-12.70)
71-80 yrs	72	0.00144	8.23	(4.65-15.95)
81+ yrs	25	0.00072	4.11	(2.11-8.47)
**T-stage**				
No BC	173	0.00068	1.00	Ref.
T0	12	0.00170	2.50	(1.32-4.29)
T1	33	0.00134	1.96	(1.33-2.81)
T2	21	0.00130	1.90	(1.18-2.92)
T3-4	2	0.00055	0.80	(0.13-2.50)
TX	2	0.00279	4.08	(0.67-12.79)
**N-stage**				
No BC	173	0.00068	1.00	Ref.
N0	47	0.00160	2.35	(1.69-3.22)
N1-3	9	0.00080	1.17	(0.56-2.16)
N4+	4	0.00077	1.13	(0.35-2.66)
NX	10	0.00154	2.26	(1.12-4.05)
**ER-status**				
No BC	173	0.00068	1.00	Ref.
ER+	56	0.00153	2.25	(1.65-3.02)
ER-	7	0.00088	1.28	(0.55-2.53)
Missing	7	0.00091	1.33	(0.57-2.62)
**PR-status**				
No BC	173	0.00068	1.00	Ref.
PR+	48	0.00157	2.30	(1.66-3.14)
PR-	14	0.00103	1.51	(0.84-2.50)
Missing	8	0.00099	1.45	(0.66-2.76)
**Surgery**				
No BC	173	0.00068	1.00	Ref.
No surgery	3	0.00139	2.04	(0.50-5.35)
BCS	44	0.00156	2.28	(1.62-3.15)
Mastectomy	21	0.00099	1.46	(0.90-2.24)
Missing	2	0.00290	4.25	(0.70-13.31)
**Endocrine therapy**				
No BC	173	0.00068	1.00	Ref.
Yes	47	0.00140	2.06	(1.47-2.81)
No	23	0.00123	1.80	(1.13-2.72)
**Chemotherapy**				
No BC	173	0.00068	1.00	Ref.

Yes	11	0.00074	1.08	(0.55-1.90)
No	59	0.00158	2.32	(1.71-3.09)
**CCI**				
0	187	0.00070	1.00	Ref.
1	31	0.00120	1.71	(1.15-2.46)
2	14	0.00178	2.55	(1.41-4.22)
3+	11	0.00235	3.36	(1.72-5.88)
**Educational level**				
low	107	0.00108	1.00	Ref.
middle	98	0.00085	0.78	(0.60-1.03)
high	34	0.00043	0.40	(0.27-0.58)
missing	4	0.00032	0.29	(0.09-0.70)
**Year of BC diagnosis**				
1992-2001	69	0.00056	1.00	Ref.
2002-2012	174	0.00096	1.72	(1.31-2.29)

Odds ratio of women with lung cancer diagnosed within one year from breast cancer (BC) diagnosis. Number (No), lung cancer (LC), Odds ratio (OR), confidence interval (CI), years (yrs), tumor status (T-stage), pathological lymph node stage (N-stage), estrogen receptor status (ER-status), progesterone receptor status (PR-status), and Charlson comorbidity index (CCI).

Supplementary Table 4: Risk of ipsi- and contralateral lung cancer in women with breast cancer between 1992 and 2001 compared to women without breast cancer diagnosis

	No. of events	Inc. per 100000 pyrs	Crude		Adjusted[#]	
			HR	95% CI	HR	95% CI
**Ipsilateral LC**						
**BC**						
No BC	328	46.7	1.00	Ref.	1.00	Ref.
BC	107	74.4	1.61	(1.29-2.00)	1.60	(1.29-1.99)
**RT**						
No BC	328	46.7	1.00	Ref.	1.00	Ref.
BC, No RT	20	47.9	1.04	(0.66-1.63)	0.93	(0.59-1.46)
BC, RT	87	85.2	1.84	(1.45-2.33)	1.92	(1.51-2.43)
**RT/N-stage**						
No BC	328	46.7	1.00	Ref.	1.00	Ref.
BC, No RT	20	47.9	1.04	(0.66-1.63)	0.95	(0.60-1.50)
BC, RT N0	54	88.1	1.89	(1.42-2.52)	1.96	(1.47-2.62)
BC, RT N1-3	21	90.5	1.96	(1.26-3.05)	2.08	(1.34-3.24)
BC, RT N4+	5	57.1	1.25	(0.52-3.02)	1.36	(0.56-3.30)
BC, RT NX	7	78.8	1.75	(0.83-3.71)	1.53	(0.72-3.25)
**Contralateral LC**						
**BC**						
No BC	345	49.1	1.00	Ref.	1.00	Ref.
BC	90	62.6	1.29	(1.02-1.62)	1.30	(1.03-1.64)
**RT**						
No BC	345	49.1	1.00	Ref.	1.00	Ref.
BC, No RT	20	47.9	0.98	(0.63-1.55)	0.89	(0.56-1.40)
BC, RT	70	68.5	1.41	(1.09-1.82)	1.49	(1.15-1.93)
**RT/N-stage**						

No BC	345	49.1	1.00	Ref.	1.00	Ref.
BC, No RT	20	47.9	0.98	(0.63-1.55)	0.92	(0.58-1.44)
BC, RT N0	40	65.3	1.33	(0.96-1.85)	1.41	(1.01-1.95)
BC, RT N1-3	13	56.0	1.15	(0.66-2.01)	1.24	(0.71-2.15)
BC, RT N4+	5	57.1	1.19	(0.49-2.88)	1.31	(0.54-3.18)
BC, RT NX	12	135.2	2.87	(1.61-5.11)	2.51	(1.40-4.48)

Hazard ratios (HR) for ipsi- and contralateral lung cancer (LC) conditioned on 5-year event-free survival. Restricted to women undergoing surgery for breast cancer (BC) and their comparison women without BC diagnosis, Number (No.), incidence (Inc.), person years (pyrs), reference (Ref.), and radiotherapy (RT). #Adjusted for age, year of BC, educational level, Chronic Pulmonary Disease (CPD), and CCI except CPD.

Supplementary Table 5: Snomed codes

Adenocarcinoma: 81403, 81443, 82303, 82502, 82503, 82533, 82573, 82603, 82653, 83333, 84803, 85513.
Squamous cell cancer: 80703, 80713, 80723, 80833.
Small cell lung cancer: 80413, 80453.
