# Supplementary material for: Risk of primary lung cancer after adjuvant radiotherapy in breast cancer—a large population-based study
Source: NPJ Breast Cancer. 2021 Jun 1;7:71. doi: 10.1038/s41523-021-00280-2 (PMC8169889; doi:10.1038/s41523-021-00280-2)
Supplement: Supplementary file 2 — Reporting Summary [file 41523_2021_280_MOESM2_ESM.pdf]

nature research

Corresponding author(s): Anna-Karin Wennstig

Last updated by author(s): 6 May 2021

Reporting Summary

Nature Research wishes to improve the reproducibility of the work that we publish. This form provides structure for consistency and transparency in reporting. For further information on Nature Research policies, see our Editorial Policies and the Editorial Policy Checklist.

Statistics

For all statistical analyses, confirm that the following items are present in the figure legend, table legend, main text, or Methods section.

n/a	Confirmed	
☐	☒	The exact sample size (n) for each experimental group/condition, given as a discrete number and unit of measurement
☒	☐	A statement on whether measurements were taken from distinct samples or whether the same sample was measured repeatedly
☐	☒	The statistical test(s) used AND whether they are one- or two-sided<br>*Only common tests should be described solely by name; describe more complex techniques in the Methods section.*
☐	☒	A description of all covariates tested
☐	☒	A description of any assumptions or corrections, such as tests of normality and adjustment for multiple comparisons
☐	☒	A full description of the statistical parameters including central tendency (e.g. means) or other basic estimates (e.g. regression coefficient) AND variation (e.g. standard deviation) or associated estimates of uncertainty (e.g. confidence intervals)
☐	☒	For null hypothesis testing, the test statistic (e.g. F, t, r) with confidence intervals, effect sizes, degrees of freedom and P value noted<br>*Give P values as exact values whenever suitable.*
☒	☐	For Bayesian analysis, information on the choice of priors and Markov chain Monte Carlo settings
☒	☐	For hierarchical and complex designs, identification of the appropriate level for tests and full reporting of outcomes
☒	☐	Estimates of effect sizes (e.g. Cohen's d, Pearson's r), indicating how they were calculated

Our web collection on statistics for biologists contains articles on many of the points above.

Software and code

Policy information about availability of computer code

Data collection	Not applicable
Data analysis	Not applicable

For manuscripts utilizing custom algorithms or software that are central to the research but not yet described in published literature, software must be made available to editors and reviewers. We strongly encourage code deposition in a community repository (e.g. GitHub). See the Nature Research guidelines for submitting code & software for further information.

Data

Policy information about availability of data

All manuscripts must include a data availability statement. This statement should provide the following information, where applicable:

- Accession codes, unique identifiers, or web links for publicly available datasets
- A list of figures that have associated raw data
- A description of any restrictions on data availability

The data that support the findings of this study are available from the corresponding author upon reasonable request

Field-specific reporting

Please select the one below that is the best fit for your research. If you are not sure, read the appropriate sections before making your selection.

☒ Life sciences ☐ Behavioural & social sciences ☐ Ecological, evolutionary & environmental sciences

For a reference copy of the document with all sections, see nature.com/documents/nr-reporting-summary-flat.pdf

Life sciences study design

All studies must disclose on these points even when the disclosure is negative.

Sample size	The Breast Cancer DataBase Sweden (BCBaSe) cohort was used. It comprises all new cases of invasive breast cancer in women from 1992 to 2012 in three Swedish health care regions, and a comparison cohort of five age-matched women without a BC diagnosis for each case of breast cancer. A total of 52300 women with breast cancer were included, and 253796 women without breast cancer.
Data exclusions	Women with metastatic breast cancer at diagnosis, a contralateral breast cancer during follow-up, or a history of another malignancy were excluded. For the Northern region, only women treated between 2008 and 2012 were included, due to inconsistencies concerning registration in the regional breast cancer register prior to 2008.
Replication	The study was a registry-based retrospective cohort study, and not an experimental study, and therefore this was not relevant.
Randomization	The study was a registry-based retrospective cohort study, and no randomisation was performed.
Blinding	The study was a registry-based retrospective cohort study, and blinding of the cohorts was therefore not relevant.

Reporting for specific materials, systems and methods

We require information from authors about some types of materials, experimental systems and methods used in many studies. Here, indicate whether each material, system or method listed is relevant to your study. If you are not sure if a list item applies to your research, read the appropriate section before selecting a response.

Materials & experimental systems

n/a	Involved in the study	
☒	☐	Antibodies
☒	☐	Eukaryotic cell lines
☒	☐	Palaeontology and archaeology
☒	☐	Animals and other organisms
☐	☒	Human research participants
☒	☐	Clinical data
☒	☐	Dual use research of concern

Methods

n/a	Involved in the study	
☒	☐	ChIP-seq
☒	☐	Flow cytometry
☒	☐	MRI-based neuroimaging

Human research participants

Policy information about studies involving human research participants

Population characteristics	The Breast Cancer DataBase Sweden (BCBaSe) cohort generated from registry data was used. BCBaSe comprises all new cases of invasive breast cancer in women from 1992 to 2012 in three Swedish health care regions, and a comparison cohort of five age-matched women without a BC diagnosis for each case of breast cancer. A total of 52300 women with breast cancer were included, and 253796 women without breast cancer.
Recruitment	Retrospective registry-based study cohort. For inclusions see above.
Ethics oversight	Regional ethics committee of in Stockholm, Sweden. Reference number 2013/1272-31/4.

Note that full information on the approval of the study protocol must also be provided in the manuscript.
